# Online medical education in Egypt during the COVID-19 pandemic: a nationwide assessment of medical students’ usage and perceptions

**DOI:** 10.1186/s12909-022-03249-2

**Published:** 2022-03-30

**Authors:** Mohamed Mortagy, Aya Abdelhameed, Patricia Sexton, Melissa Olken, Mohamed Tharwat Hegazy, Mohammed Abdel Gawad, Fathy Senna, Islam A. Mahmoud, Jaffer Shah, Egyptian Medical Education Collaborative Group (EGY MedEd), Abdelrahman Elkholy, Abdelrahman Mahmoud, Ahmad Elframawy, Ahmed Emara, Ahmed Abualez, Ahmed Naeem, Ayda Mohamed, Bishoy Fahim, Diaa Saadeh, Hana Yehia, Hisham Alsharif, Hossam Ali, Karim Khalil, Karim Sayed, Mark Farag, Mohamed Abugdida, Pishoy Sydhom, Raafat Yousuf, Rajya Ahmed, Ramadan Farahat, Rana Elbayar, Rowaina Diab, Yousra Hussein, Hani Aiash

**Affiliations:** 1 Internal Medicine Department, NewGiza University School of Medicine, Giza, Egypt; 2 Egyptian Medical Education Collaborative Group (EGY MedEd), Cairo, Egypt; 3 Alexandria University Faculty of Medicine, Alexandria, Egypt; 4 Family Medicine Department, AT Still University, Kirksville College of Osteopathic Medicine, Kirksville, USA; 5 Internal Medicine Department, Western Michigan University Homer Stryker M.D. School of Medicine, Kalamazoo, USA; 6 Internal Medicine Department, Faculty of Medicine, Cairo University, Cairo, Egypt; 7 Nephrology Department, NewGiza University School of Medicine, Giza, Egypt; 8 Helwan University Faculty of Medicine, Helwan, Egypt; 9 Assiut University Faculty of Medicine, Asyut, Egypt; 10 Medical Research Center, Kateb University, Kabul, Afghanistan; 11 New York State Department of Health, Albany, NY USA; 12 Department of Cardiovascular Perfusion, State University of New York Upstate Medical University, Syracuse, New York, USA; 13 Department of Family Medicine, Faculty of Medicine, Suez Canal University, Ismailia, Egypt

**Keywords:** Medical education, Online medical education, COVID-19, Medical students, Medical schools, Egypt

## Abstract

**Background:**

The coronavirus (COVID-19) pandemic required a transformation of medical education in Egypt. Public health measures necessitated a rapid shift from traditional face to face lectures to largely online platforms following campus closures. The aim of this study is to characterize medical student use and perception of online medical education in Egypt as well as exploring the efficacy of different e-learning modalities. Additionally, many barriers and opportunities as perceived by students are reviewed to inform future educational improvements.

**Methods:**

A 29-item online survey was created on google forms and distributed by social media to medical students across 26 Egyptian medical schools. The survey was administered from August 20th, 2021, to September 5th, 2021. The survey consisted of a mixture of questions style. The medical students were asked about their experiences with online medical education during the COVID-19 pandemic as well as medical students’ anxiety, perceived academic performance, and obstacles related to online education.

**Results:**

Of the 4935 responses collected, 43.4% (*n* = 2140) of respondents were women; 56.6% (*n* = 2795) were men. Medical students from private medical schools were 13.0% (*n* = 644), whereas 87.0% (*n* = 4291) were from public medical schools. 54.6% of students reported that online education is not as effective as face-to-face education. There was a significant rise in hours spent by medical students on online medical education compared to before COVID-19 pandemic. More than half of students (63%) agreed that online recorded video tutorials (e.g., YouTube) were the most effective form of online medical education.

**Conclusion:**

The shift to online education has significantly impacted medical students in Egypt. Medical students reported various limitations and challenges of online medical education, which must be addressed considering the potential benefits of online platforms over traditional face to face learning. The results of this nationwide study provide a framework for potential areas to implement change to improve the accessibility and structure of online medical education in Egypt.

**Supplementary Information:**

The online version contains supplementary material available at 10.1186/s12909-022-03249-2.

## Background

COVID-19 has caused devastating loss of human life, unprecedented economic impacts and social disruption touching every sector. To slow the spread of the virus, many countries imposed strict lockdowns, essentially stopping the normal course of human interaction. While no industry was spared, educational systems saw well over a billion students worldwide locked out of the face-to-face classroom [1]. In medical education, this presented as a drastic shift from primarily in-person lectures, tutorials, skills development, and clinical experiences to a variety of online learning modalities.

E-learning, defined as delivery of learning experiences through the internet, has been investigated as an effective modality for medical education for over 20 years [2, 3]. Proponents have discussed that e-learning technology gives the student control over content, pace, and the ability to tailor material to their interest [4]. It has been thought that this is integral to developing self-directed, life-long learners, which is necessary for medical professionals. Further, some assert that familiarity and comfort with this type of learning will prepare future practitioners for medical care delivery through tele-health and increase comfort with electronic health records [5]. However, a carefully designed, integrated e-learning curriculum requires time and planning [6]. Because of the abrupt onset of the Covid crisis, most schools simply placed a compilation of educational content on an electronic platform [7, 8]. That is why it was necessary to study the status and implications of online teaching on medical students’ learning.

A recent meta-analysis reviewing medical education studies from 1990 to 2019 compared blended (e-learning in combination with traditional classroom-based learning) and synchronous face-to-face learning [6]. These authors found that the extant literature demonstrates increased effectiveness of blended programs. This calls the community to investigate best practices in medical education and evaluate what aspects (exploratory sciences, clinical skills, patient care, etc.) are best done face-to-face, and what can be delivered via alternative methods. While many have supported a future of e-learning in medical education, COVID-19 caused a grand and abrupt trial of this approach. Researchers and students alike have noted both opportunities and barriers that arise using online platforms [9–11].

Palvia, et al. reviewed the worldwide status of e-learning in 2018 and found that, the use of technology is only expected to increase in the medical education realm throughout the next decade. Internationally, the implementation of educational technology varies, with some markets, including the Middle East, having lower penetrance and lower public acceptance [12, 13]. The literature is ripe with examples both supporting e-learning for medical education citing student benefits such as flexibility and increased student control but also noting concerns about use of e-learning for acquisition of clinical skills [14–17]. Others have presented a comparative analysis of online versus conventional learning that found that conventional teaching was preferred by students [18]. This study revealed student and staff concerns regarding technology, accessibility, and cheating. There are many published guides for review and evaluation of online learning processes, however, in the current situation, no institution had the time to appropriately plan for the abrupt move to pure e-learning [11]. Gaps include lack of educational infrastructure including needed modifications to learning management systems, the need for high quality learning materials (videos, taped lectures, etc.), the ability to deliver student assessment that assures exam security and validity, and, finally the lack of faculty trained to facilitate online learning. Additionally, even prior to the pandemic researchers published cries to revisit the attributes that make a physician successful in-patient care – and to look beyond acquisition of scientific knowledge alone [19]. The gap in clinical skills acquisition and professional identify formation may cause issues for the cohorts of students educated with less hands-on instruction during the COVID lockdowns [20].

The aims of this study were to explore efficacy of different e-learning modalities, as well as personal and professional opportunities or barriers as perceived by medical students in Egypt including teacher preparedness and medical school adaptations during the covid 19 pandemic. This is important, given the relative resource constraints in low- and middle-income countries, including Egypt [21]. To our knowledge, this is the first nationwide assessment of medical student’s use and perception of online medical education in Egypt during the COVID-19 pandemic. The pandemic did not provide institutions the time to strategize, develop faculty, nor invest in infrastructure. Lessons learned from the present study will inform improvement of medical education in Egypt and preparedness for future crises.

## Methods

### Questionnaire design and distribution

This was a cross sectional study conducted on a national level in Egypt via an online survey disseminated through social media channels (Facebook, WhatsApp, Telegram and emails). A 29-item questionnaire was devised based on published literature. Questions addressing the experiences of online teaching were based on the Dundee Ready Education Environment Measure (DREEM), a validated questionnaire that is designed to measure the educational environment of medical schools and healthcare professionals [22]. Our questionnaire added some questions addressing medical student’s anxiety levels. The questionnaire was also based on a recent national study done in the United Kingdom (UK) that addressed the same topic [23]. Ethical approval was taken from New Giza University School of Medicine’s ethics committee.

The questions were initially drafted from these 2 sources and discussed with a pilot sample of Egyptian medical students and medical educators before undergoing review and final editing. The remaining items in the questionnaire comprised a mixture of questions style including multiple choice questions, checklist choice questions, fill in the blank questions and 5- point Likert scale questions. The final questions addressed four main themes:

1. General demographics
2. The use and perception of online medical education before and during COVID-19 pandemic
3. The benefits and barriers of online medical education
4. The impact of online medical education on medical students’ anxiety and perceived level of academic performance.

The survey was created using an online google form and distributed by a medical student collaborative group that was recruited nationally with a representative in most Egyptian medical schools (Egyptian Medical Education Collaborative Group;EGY MedEd). The survey was distributed from the 20th of August 2021 till the 5th of September 2021 to Egyptian medical students through social media.

### Participants

All medical students in Egyptian medical schools across different academic years (1st year through 7th year) were eligible to participate. Egypt has 30 registered medical schools in the world directory of medical schools (WDOMS). The total number of medical students in Egypt is estimated to be around 70,000 medical students.

### Data analysis

Data were exported from google form to google sheets and then to Microsoft excel sheet (excel V 16.0, 2021). Graphs, figures and descriptive statistics were produced using R language and R Studio (version 4.1.1). Shapiro-Wilk test showed that the number of hours of online medical education spent by medical students before and during the covid-19 pandemic were non-normally distributed. Therefore, Wilcoxon test was used for their statistical analysis. *P* values < 0.05 were considered significant.

## Results

### Cohort demographics

Cohort demographics table is available in supplementary data table. Of the 4935 responses collected, 43.4% (*n* = 2140) of respondents were women; 56.6% (*n* = 2795) were men. Responses were collected from 26 medical schools across Egypt, 13.0% (*n* = 644) were private schools, 87.0% (*n* = 4291) were public. They were collected from medical students across all years: The majority of respondents were from year 4 (17.3%) and 5 students (17.6%) while the minority of students were from year 7 (7.2%). It is important to note that the rules of admissions, tuition fees and examination cut off scores vary between private and public Egyptian medical schools [24]. Tuition fees for private medical schools are higher than public medical schools. However, required examination cut off scores are less for public medical schools.

### Use and perception of online medical education before and during COVID-19 pandemic

**Fig. 1:**
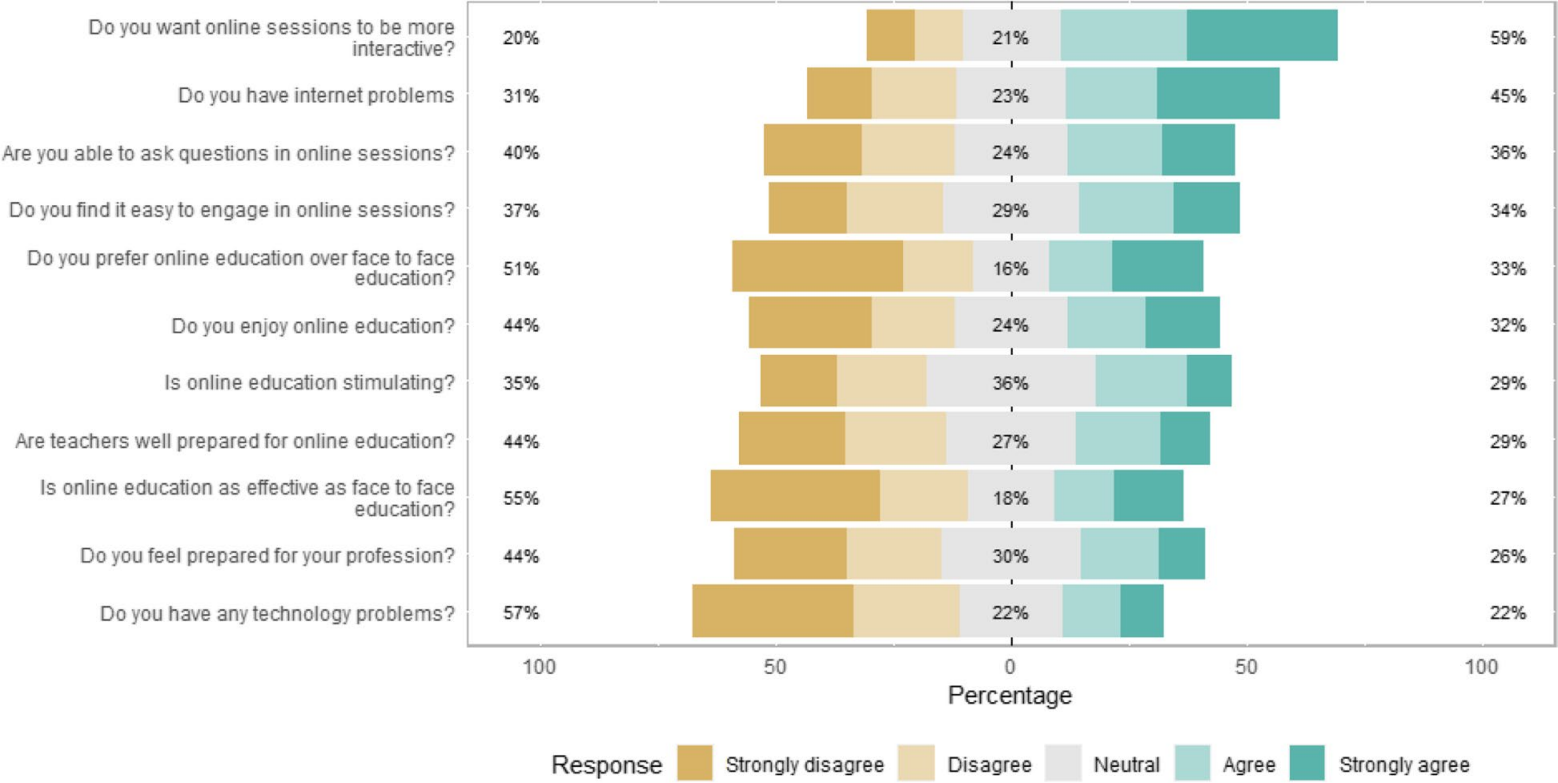
Perception of online medical education among medical students in Egyptian medical schools during the COVID-19 pandemic

Perception of online medical education during COVID-19 is shown in Fig. 1. Some responses to several questions varied a great deal among students. Majority of students (64.6%) perceived that online education is better than over face-to-face education However, many of the students (51%) preferred face to face education over online education as their modality of choice for medical education. Many of the students (44.1%) reported that their teachers were not well prepared for online education. A great proportion of students (45.2%) reported having internet problems such as internet connection issues and/or internet speed. A majority of students (57%) denied having technology use problems such as personal difficulties to use online learning management system or attending online teaching sessions. More than 50% of students wanted online sessions to be more interactive and 44% felt that they are not prepared for their profession, and this was a consistent percentage across all academic years.

Some students (35%) reported that online medical education is non-stimulating as 37.3% of students had difficulty engaging in online sessions and 40.7% of students didn’t feel that they are able to ask questions freely in online sessions. These factors may reflect why only 43.7% of medical students enjoyed online medical education.

Comparison between hours spent by Egyptian medical students on online medical education before and during the COVID-19 pandemic is shown in Table 1. There was a significant rise in hours spent by medical students on online medical education during COVID-19 pandemic compared to hours spent before the pandemic. Mean hours spent during the pandemic were 20.69 versus 11.23 h spent before the pandemic (*p* value < 0.05).

**Table 1:**
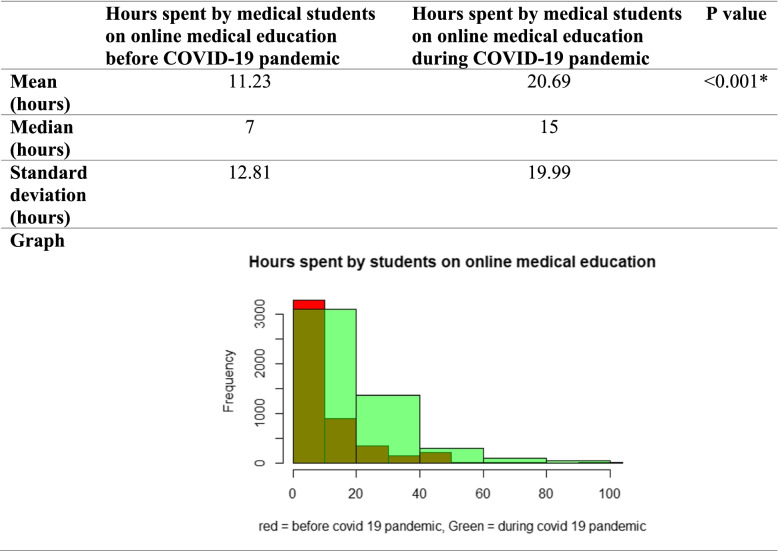
Comparison between hours spent by medical students on online medical education before and during the COVID-19 pandemic

* Significant (*p* value < 0.05)

### Medical school adaptations and form of interaction during online sessions (Fig. 2A, B)

The adaptation of medical schools to COVID-19 in the lockdown to continue the teaching process appeared in many ways (Fig. 1). Students were asked about medical school adaptations in response the covid-19 pandemic and lockdown. These adaptations included teaching delivery modalities (e.g., live sessions delivery with ZOOM), ability to reach online resources and the availability of online resources that were provided by their medical schools (e.g., learning management system and other online resources).

**Fig. 2:**
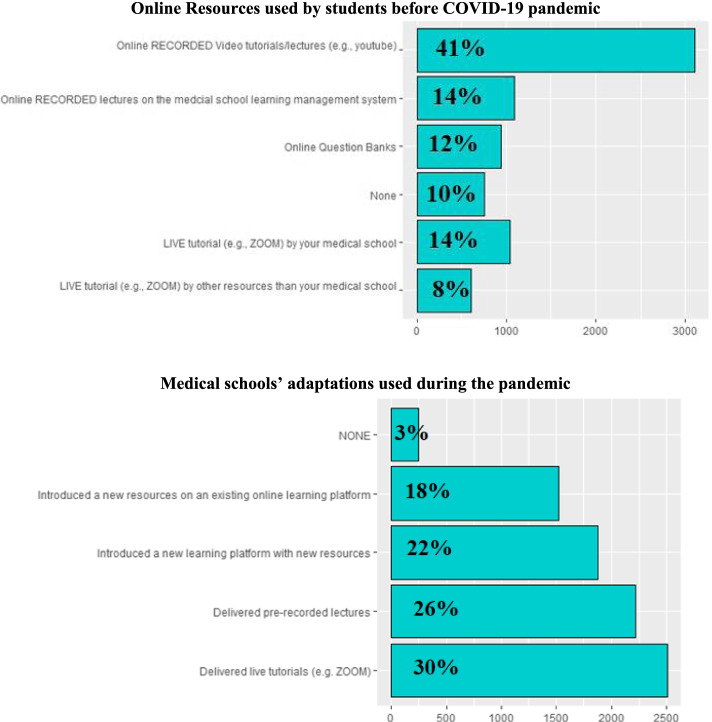
**A**, **B** Online resources used by students before COVID-19 pandemic and medical schools’ online adaptations used during the pandemic

During the pandemic, some students (30%) received live tutorials whereas 26% received pre-recorded lectures as a part of their medical school adaptations. Before the pandemic, some students (14%) received live tutorials and 14% received pre-recorded lectures from their medical schools. Some students (22%) reported that their medical schools introduced a new learning platform with new resources and 18% reported that their medical schools added new resources on an existing online learning platform during the pandemic. Students who didn’t use any online resources whether live or pre-recorded lectures were a minority (10%) before the pandemic.

**Fig. 3:**
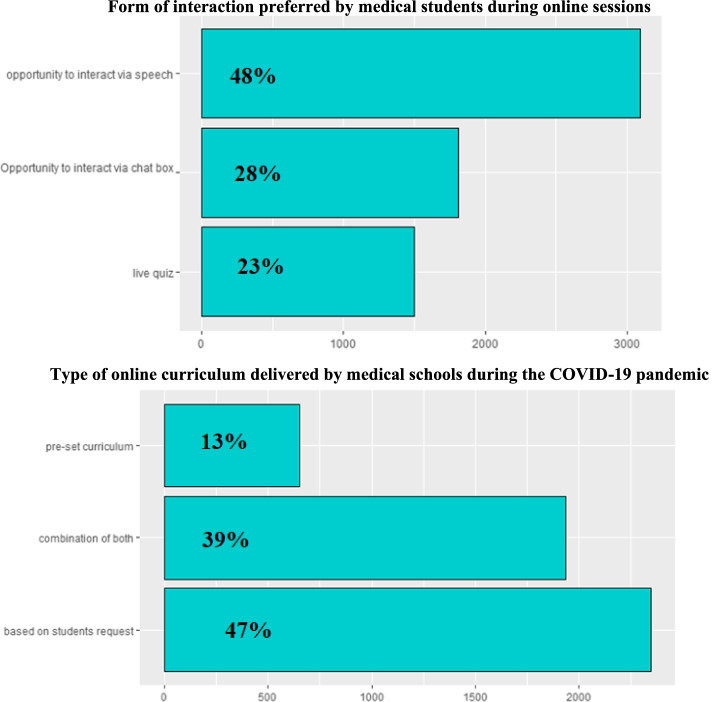
**A,** **B** Form of interaction preferred by medical students during online sessions and type of online curriculum delivered by medical schools during the COVID-19 pandemic

The form of interaction preferred by medical students during online sessions and type of online curriculum delivered by medical schools during the COVID-19 pandemic are shown in Fig. 3A, B. Many students (48%) preferred online interaction via speech as their preferred method of communication whereas 28% preferred chat box and 23% preferred live quizzes.

Curriculum delivery varied by Egyptian medical schools during the pandemic. Some students (47%) reported that the live online sessions delivered by medical schools were based on student’s request (e.g., difficult topics) relied on pre-recorded lectures or self-study for the rest of the curriculum. Only a small portion of students (13%) reported that their medical schools delivered online live sessions according to a pre-set curriculum and not based on students’ choice. Some students (39%) reported that their medical schools used a combination of both modalities.

### The benefits and barriers of online medical education and effective forms of medical education

**Fig. 4:**
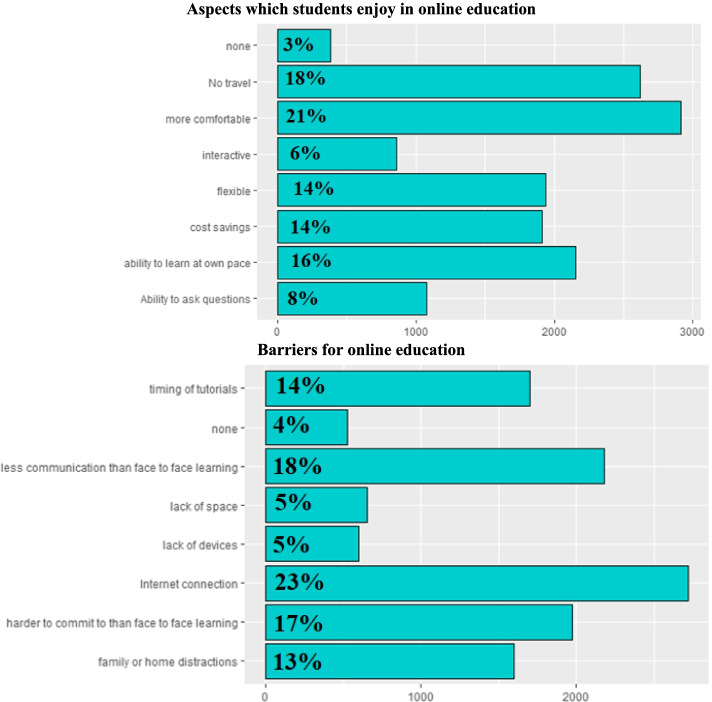
**A**, **B** Aspects which students like and dislike in online education

The aspects which students enjoy in online education are shown in Fig. 4A. Some students (21%) agreed that online education is more comfortable as they don’t need to commute or travel to the medical school, and it is easier to receive lectures from the comfort of being in their houses. Also, 16% enjoyed the benefit of the ability to learn at their own pace while studying at home, 14% found online education more flexible and 14% found it cost saving.

On the contrary, some students reported many barriers for online education (Fig. 4B) as 23% complained of bad internet connection., 17% felt it was difficult to commit to studying than face to face learning because of home distractions and 18% perceived less communication in comparison face to face learning.

**Fig. 5:**
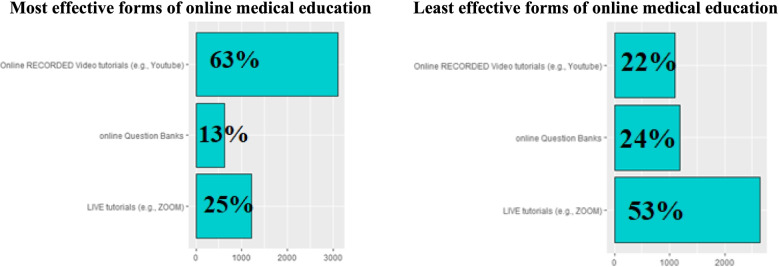
**A**, **B** Most and Least effective forms of online medical education

The most and least affective aspects of online medical education are shown in Fig. 5A, B. More than half of students (63%) agreed that online recorded video tutorials through YouTube are the most effective aspect of online medical education. However, only 25% admitted that live tutorials through Zoom are most effective. A minority (13%) found online question banks most effective.

### Impact of online medical education on medical students’ anxiety and perceived level of academic performance

**Fig. 6:**
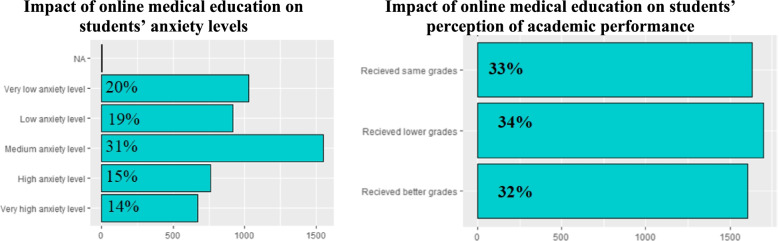
**A**, **B** Impact of online medical education on student anxiety levels and their perception of academic performance

The impact of online medical education on medical students’ anxiety and perceived level of academic performance is shown in Fig. 6A, B. Some students (31%) reported medium anxiety level. Many reported low (19%) and very low anxiety levels (20%). Smaller proportion reported high (15%) and very high anxiety levels (14%). Students were asked about their perception of the effect of COVID 19 pandemic on their academic performance reflected by their current grades compared to their grades before the pandemic. Students who reported lower grades (34%) were almost equal to students who reported the same (33%) or better grades (32%).

## Discussion

### Impact of COVID-19 pandemic on online teaching worldwide

Nearly every medical school in the world has been affected by the COVID-19 pandemic. From disruption of classroom learning and traditional clinical skills building to cessation of hands-on clinical experiences due to lack of personal protective equipment, medical students and their faculty across the globe have been faced with abrupt changes to curriculum and training delivery [23]. Responses to the educational disruptions ranged from short-term cessation of courses to social isolation using on-campus facilities, to rapid movement of curriculum to electronic offerings. Internationally, some institutions had relatively robust learning management systems, internet capabilities, and trained faculty that enabled seamless transitioning to an online environment. Other medical schools struggled to create digital content, train faculty, and develop infrastructure to support such a move [13–17]. A cross-country comparison of the impact of COVID-19 on higher education in general demonstrated that developed economies primarily moved higher education online while developing economies closed schools for at least some period of time [25]. A large cross-sectional study of UK medical students’ perceptions toward online teaching during the pandemic indicated that, although the educational process moved forward, students were less satisfied with online education and expressed concerns for their clinical preparedness [23]. This theme is born out in medical schools across the Middle East. A study in Jordan indicated that, at least early distance learning attempts were deemed unsatisfactory by students [16]. Several barriers were identified including those pertaining to technology (internet access and quality). However, these students believe, that some form of blended learning is likely to remain after the pandemic is controlled. In Libya, less than 30% of medical students responding to a study survey had participated in formal medical education coursework via e-learning prior to the pandemic [15]. Most of that same study group (64.7%) disagreed that e-learning can be implemented in Libya. In Egypt, the pandemic has presented an opportunity for better utilization of national learning resource repositories such as the Egyptian Knowledge Bank (EKB), an online library of learning resources offered free-of-charge to all Egyptian citizens, established by the Egyptian government in 2016 [17]. Finally, Several studies note that students had disruptions in hands-on clinical training and, as such, have increased concerns about their preparedness for patient care [26–28]. These concerns, along with the fear of contracting COVID-19 themselves or exposing their families, has led to psychological impacts on these students [28]. The current study aligns well with published data. Students in Egyptian medical schools reported that faculty may not be fully prepared for online teaching, as well as concerns regarding preparedness for the clinical setting. However, as this study was completed over a year from the onset of the pandemic, and thus a year after many educational adaptations, most of these students found e-learning to be as effective as face-to-face teaching, fewer issues with technology and slightly less anxiety. Additionally, they reported positive aspects such as no travel, more comfort, and learning at their own pace.

### Assessment of online infrastructure in the Middle East

Egypt is undergoing major educational changes due to the implementation of The Egyptian presidential program, which will be completed by 2030. This program includes the introduction of a wide range of technology including locally manufactured tablets and laptops to be used by the students. This program also includes the Egyptian Knowledge Bank (EKB), that was established in 2016, it provides information and resources needed by Egyptian researchers and students. It is a cornerstone for e-learning for undergraduate and post-graduate students and researchers [17, 29].

Other countries in the Middle East have different infrastructures and facilities for e-learning. During the COVID crisis most of the public universities in the Middle East and North Africa (MENA) region struggled with the sudden need for online teaching after the lock down [30, 31].

Online medical education is faced with many challenges. First, the defect in the infrastructure due to lack of access to stable internet on wide scale, lack of hardware such as tablets and laptops for student use at home, and lack of online learning platforms. Second, limited availability of materials and technology for online courses. Third, limited social interaction, without face-to-face learning, which is a challenge especially in medical teaching [31].

Despite these challenges, MENA countries have adapted to cope with this crisis. They moved most of their courses online and through online platforms using different programs as ZOOM, Microsoft teams, and TV channels dedicated to broadcasting lectures. Some countries have cancelled or postponed exams (online exams or project-based assignments) [31].

### Student perception of online teaching

Some drawbacks to online teaching were identified in our study. Most respondents disagreed that online education is an interesting method and claimed that online teaching is not as effective as face-to-face teaching Many of them found that online teachers were not well prepared for the online sessions. The shift towards online education happened quickly without the opportunity for adequate faculty development and faculty adjustments to teaching materials or styles. Students were neutral towards the ease of their engagement and their ability to ask questions in addition to finding online method stimulating. However, in another study [32], students were frustrated and constrained by limited options in online teaching as they suffered lack of meaningful connections and their faculty relied heavily on online teaching. Students in that study reported that they were not getting the same chance to learn and complained of little privacy for both faculty and students. In addition, their opinion was that online teaching lacks collaboration which makes it non-reliable and non-effective in terms of brainstorming and critical thinking, and their sense of guidance and support. They also complained of their waste of time on technology.

Many students in another study perceived online method as a time saving method [18]. Van de Vord et al. [33] confirmed this point by observing that the total teaching time per week per student spent by online teachers was significantly shorter in online teaching.

Students noted important practices that should be focused for better online learning. In our study, most suggested that online method could be more interactive. In another study [15], students suggested that regular formative assessment could accomplish more student engagement. Other students in the same study suggested using games, quizzes, and multimedia for the online classes to be more interesting. For the development of clinical reasoning skills, some students suggested the incorporation of scenarios, interactive diagnostic reasoning software and virtual simulation. Another point that students mentioned was the student-centered activities that help motivate students and let students to be more cooperative. They suggested the creation of online team-based learning (TBL) for better collaboration. A recent systematic review done on 39 articles suggested that virtual medical teaching during the covid 19 crisis is effective [34].

### Benefits and barriers of online teaching, its paradox and possible explanation

Students perceived many benefits with on-line learning. Most students appreciated the conveniences of learning from home. It was more comfortable, did not involve travel, nor the expense of travel. For asynchronous events, students had the flexibility of learning at their own pace and choosing when they learned. Most students preferred pre-recorded videos and lectures and most of them engaged with online platforms even before the pandemic. However, most students did not find on-line learning enjoyable or even liked it as an alternative to face-to-face teaching. This paradox may reflect the barriers inherent in on-line learning.

The theme of perceived problems with on-line learning revolved around the ability of students to interact with the instructor and each other. A sense of “professional community” is lost. Students found it difficult to be engaged with the lessons citing internet connectivity problems as well as distractions in their home environments. Types of distractions were family, home responsibilities and tasks, and time commitment. Self-regulated time management is essential for students to login to synchronous events and plan when they will view asynchronous sessions. These barriers were likely not uniform across students surveyed. Students with poor internet access, large families, and different noise levels (e.g., rural vs urban) may be more adversely impacted.

Many reasons may explain these barriers including the rapid shift towards online teaching and the unprepared teachers for this new situation. The COVID-19 pandemic forced medical schools to urgently transform classroom, lab, and clinical skills learning events to an on-line environment. With the “emergency” nature of transitioning to on-line learning, many institutions may not have had the information technology infrastructure to have this happen as effectively and quickly as desired [35, 36] Further, faculty who had less experience teaching on-line may not have had faculty development opportunities to help them transform their material. Therefore, it is premature and unjust to judge online medical education given these circumstances and this could be explored in future studies.

### Impact of online teaching on medical students’ anxiety

Our study showed that many students reported varying levels of anxiety. Aidos et al. conducted a questionnaire-based cross-sectional study among medical students ranging from 1st year to 5th year at Astana Medical University, Turkey [37]. The findings revealed that prevalence of burnout, depression, anxiety, and somatic symptoms decreased after transitioning from traditional learning to online learning during the pandemic period of COVID-19. Among students who indicated a decrease in academic performance during online learning, depression and anxiety symptoms, dissatisfaction with academic performance were the most common drawbacks. Students who lived alone during the quarantine were more prone to depression during online learning.

A descriptive study for all medical students at the American University of Beirut Faculty of Medicine was done by Bachir et al. [30] The majority of medical students reported that they felt more stressed after shifting to online classes. Medical students also reported that they would be willing to go back to on-campus classes. In our study, many students (44%) felt unprepared for their profession which might contribute to their anxiety levels.

A cross-sectional study was conducted at six Jordanian medical schools and found that about half of the participants had a severe mental disorder, and only 13.2% were likely to be well [38]. The study indicates that half of the medical students suffered mental disorders during the COVID-19 pandemic. Our study did indicate some student anxiety.

### Impact of online teaching on medical students’ academic performance

Egyptian medical students indicated no trend in any direction regarding perceived impact of online education. Approximately equal numbers indicated their grades were the same, lower, or higher as compared to if they had been in face-to-face education. The literature is inconclusive on this issue. One cross sectional study observed that student performance increased with the online method [39]. Another randomized controlled trial study that was conducted to compare between 3D virtual classroom versus traditional classroom on abdominal radiology education concluded that both ways were similar in clinical skills acquisition [40].

### Future directions of online teaching

Because medicine is an intensely interpersonal field, requiring advanced skill and the ability to work in teams as well as with patients, a pure online approach may not be suitable. Institutions must learn from the lessons of the COVID-19 lockdown how to make education more efficient and effective while maintaining the quality of physician training so as not to put society at risk. We have many recommendations for making online medical education more effective and efficient. First, training faculty on delivering online live and recorded sessions in an interesting and efficient way. Second, upgrading and installing new modern technological infrastructure at medical schools for online learning. Lastly, taking into account student perception and suggestion to improve the student’s satisfaction which is necessary for their motivation and career preparedness.

### Limitations

Although the current study is a descriptive study that uses the perceptions of students in different Egyptian Universities, the findings must be interpreted with caution.

Our study didn’t include analysis of institutional factors or feedback from faculty which are needed to improve medical education in the future. The number of students shared in the survey is not an accurate representative to the total number of students in each University. Also, students described their personal experiences in different provinces of Egypt with variable socioeconomic classes. It is also important to note the sample size in the current study while interpreting the study findings. Focus groups would have been beneficial to gain further insight into the data obtained from the survey.

## Conclusion

Online Medical Education existed to a small degree in many medical schools in Egypt before COVID-19 pandemic. However, during the COVID-19 pandemic, many medical schools were forced to implement a full online medical education model rapidly, without having the appropriate infrastructure or experience to deliver high quality medical education in this format. This was reflected by the perception of medical students in this study. Online medical education is promising due to its benefits, but it has many barriers that we must face and overcome first. Certainly, more research and development need to be done in this field. We believe that more research has to be done periodically to evaluate and assess the medical students’ experience once new changes are implemented in the online medical education sector and to assess the long-term outcomes of online medical education.

## Supplementary Information

**Additional file 1.**

**Additional file 2.**

## Data Availability

The anonymized datasets used and/or analyzed during the current study are available from the corresponding author on reasonable request.

## Abbreviations

WDOMS: World directory of medical schools
TBL: Team-based learning
MENA: Middle East and North Africa
UK: United Kingdom
