# Supplementary material for: Online medical education in Egypt during the COVID-19 pandemic: a nationwide assessment of medical students’ usage and perceptions
Source: BMC Med Educ. 2022 Mar 30;22:218. doi: 10.1186/s12909-022-03249-2 (PMC8966850; doi:10.1186/s12909-022-03249-2)
Supplement: Supplementary file 1 — Additional file 1. [file 12909_2022_3249_MOESM1_ESM.docx]

**Supplementary Data**

**Survey respondents’ demographic data**

|  | **Female (N=2139)** | **Male (N=2796)** | **Overall (N=4935)** |
| --- | --- | --- | --- |
| **Medical School Name** |  |  |  |
| 6th of October University | 49 (2.3%) | 98 (3.5%) | 147 (3.0%) |
| Ain Shams University | 135 (6.3%) | 157 (5.6%) | 292 (5.9%) |
| Alexandria University | 287 (13.4%) | 241 (8.6%) | 528 (10.7%) |
| Armed Forces University | 0 (0%) | 7 (0.3%) | 7 (0.1%) |
| Assiut University | 286 (13.4%) | 301 (10.8%) | 587 (11.9%) |
| Aswan University | 9 (0.4%) | 11 (0.4%) | 20 (0.4%) |
| Azhar University | 58 (2.7%) | 386 (13.8%) | 444 (9.0%) |
| Benha University | 31 (1.4%) | 83 (3.0%) | 114 (2.3%) |
| Beni-Suef University | 4 (0.2%) | 11 (0.4%) | 15 (0.3%) |
| Cairo University | 147 (6.9%) | 242 (8.7%) | 389 (7.9%) |
| Fayoum University | 7 (0.3%) | 2 (0.1%) | 9 (0.2%) |
| Helwan University | 70 (3.3%) | 121 (4.3%) | 191 (3.9%) |
| Kafr Elsheikh University | 69 (3.2%) | 56 (2.0%) | 125 (2.5%) |
| Mansoura University | 233 (10.9%) | 250 (8.9%) | 483 (9.8%) |
| Menoufia University | 19 (0.9%) | 57 (2.0%) | 76 (1.5%) |
| Minia University | 20 (0.9%) | 133 (4.8%) | 153 (3.1%) |
| Misr University | 136 (6.4%) | 117 (4.2%) | 253 (5.1%) |
| Nahda University | 1 (0.0%) | 2 (0.1%) | 3 (0.1%) |
| New Giza University | 60 (2.8%) | 34 (1.2%) | 94 (1.9%) |
| Port Said University | 70 (3.3%) | 48 (1.7%) | 118 (2.4%) |
| Qena/South valley University | 58 (2.7%) | 139 (5.0%) | 197 (4.0%) |
| Sohag University | 101 (4.7%) | 61 (2.2%) | 162 (3.3%) |
| Suez Canal University | 97 (4.5%) | 64 (2.3%) | 161 (3.3%) |
| Tanta University | 45 (2.1%) | 71 (2.5%) | 116 (2.4%) |
| Zagazig University | 103 (4.8%) | 72 (2.6%) | 175 (3.5%) |
| Another university | 44 (2.1%) | 32 (1.1%) | 76 (1.5%) |
| **Academic Year** |  |  |  |
| 1st Year | 258 (12.1%) | 299 (10.7%) | 557 (11.3%) |
| 2nd Year | 400 (18.7%) | 430 (15.4%) | 830 (16.8%) |
| 3rd Year | 303 (14.2%) | 440 (15.7%) | 743 (15.1%) |
| 4th Year | 391 (18.3%) | 461 (16.5%) | 852 (17.3%) |
| 5th Year | 329 (15.4%) | 541 (19.3%) | 870 (17.6%) |
| 6th Year | 304 (14.2%) | 426 (15.2%) | 730 (14.8%) |
| 7th Year | 154 (7.2%) | 199 (7.1%) | 353 (7.2%) |
| **University Type** |  |  |  |
| Private | 317 (14.8%) | 327 (11.7%) | 644 (13.0%) |
| Public | 1822 (85.2%) | 2469 (88.3%) | 4291 (87.0%) |
