# Supplementary material for: Online medical education in Egypt during the COVID-19 pandemic: a nationwide assessment of medical students’ usage and perceptions
Source: BMC Med Educ. 2022 Mar 30;22:218. doi: 10.1186/s12909-022-03249-2 (PMC8966850; doi:10.1186/s12909-022-03249-2)
Supplement: Supplementary file 2 — Additional file 2. [file 12909_2022_3249_MOESM2_ESM.pdf]

Perception Of Online Teaching During COVID 19 Pandemic And Its Impact on Academic Performance and Anxiety levels among Egyptian Medical Students: A National Survey

Thank you so much for your interest in filling this survey which investigates the perception of online teaching during COVID 19 pandemic and its impact on academic performance an anxiety levels among Egyptian medical students

The collected data is non-identifiable and will be used for research purposes only

* Required

1. Please tick this box to proceed *

Mark only one oval.

( ) I consent to my non-identifiable information being used for research purposes and that this is my first time completing this survey

Background Information

2. Which Egyptian Medical School do you attend *

Mark only one oval.

- Ain Shams University
- Azhar University
- Alexandria University
- Armed Forces University
- Assiut University
- Aswan University
- Beni-Suef University
- Cairo University
- Benha University
- Damietta university
- Fayoum University
- Helwan University
- Kafr Elshekh University
- Mansoura University
- Menoufia University
- Minia University
- Misr University
- Nahda University
- NewGiza University
- 6th of october University
- Port Said University
- Qena/South valley University
- Sohag University
- Suez Canal University
- Tanta University
- Zagazig University
- Other University

3. What year are you in currently? *

Mark only one oval.

- ( ) First year
- ( ) Second Year
- ( ) Third Year
- ( ) Fourth year
- ( ) Fifth year
- ( ) Sixth year
- ( ) Seventh Year

4. Is your medical school public or private? *

Mark only one oval.

- ( ) Public
- ( ) Private

5. What is your gender? *

Mark only one oval.

- ( ) Male
- ( ) Female

Online Learning and Medical Education

6. Which online learning platforms/resources did you use BEFORE the COVID 19 pandemic? *

Check all that apply.

- [ ] Online RECORDED Video tutorials/lectures (e.g., youtube)
- [ ] LIVE tutorial (e.g., ZOOM) by your medical school
- [ ] LIVE tutorial (e.g., ZOOM) by other resources than your medical school
- [ ] Online Question Banks
- [ ] Online RECORDED lectures on the medcial school learning management system
- [ ] None of these options apply

7. Which Method of online learning do you find the MOST effective? *

Mark only one oval.

- ( ) Online RECORDED Video tutorials (e.g., Youtube)
- ( ) LIVE tutorials (e.g., ZOOM)
- ( ) online Question Banks

8. Which Method of online learning do you find the LEAST effective? *

Mark only one oval.

- ( ) Online RECORDED Video tutorials (e.g., Youtube)
- ( ) LIVE tutorials (e.g., ZOOM)
- ( ) online Question Banks

9. How many hours per week did you spend on online learning BEFORE the COVID Pandemic? (number only) *

____

10. How many hours per week did you spend on online learning DURING the COVID Pandemic? (number only) *

11. How has your medical school adapted teaching for you during the COVID 19 Pandemic? *

Check all that apply.

- [ ] Introduced a new learning platform with new resources
- [ ] Introduced a new resources on an existing online learning platform
- [ ] Delivered live tutorials (e.g. ZOOM)
- [ ] Delivered ore-recorded lectures
- [ ] NONE

12. Are these online sessions interactive? *

Mark only one oval.

- ( ) Yes
- ( ) No
- ( ) most of them are interactive
- ( ) most of them are not interactive

13. What form interaction do you prefer during online interactive sessions? *

Check all that apply.

- [ ] Opportunity to interact via chat box
- [ ] opportunity to interact via speech
- [ ] live quiz

14. Does the online learning follow a pre-set curriculum or is it based on students request? *

Mark only one oval.

- pre-set curriculum
- based on students request
- combination of both

Student perception of online teaching

Please rank the following statements on your experience of online learning on a scale from 1 - 5 (1= strongly disagree, 5 = strongly agree)

15. Online Teaching is stimulating most of the times *

Mark only one oval.

1	2	3	4	5

16. I find it easy to engage in the lesson *

Mark only one oval.

1	2	3	4	5

17. I feel able to ask the question that I want *

Mark only one oval.

1	2	3	4	5
⬭	⬭	⬭	⬭	⬭

18. I enjoy the online teaching *

Mark only one oval.

1	2	3	4	5
⬭	⬭	⬭	⬭	⬭

19. I would like the online teaching to be more interactive *

Mark only one oval.

1	2	3	4	5
⬭	⬭	⬭	⬭	⬭

20. I feel that the online teaching is as effective as face-face teaching *

Mark only one oval.

1	2	3	4	5
⬭	⬭	⬭	⬭	⬭

21. I prefer online teaching to face to face teaching *

Mark only one oval.

	1	2	3	4	5
	⬭	⬭	⬭	⬭	⬭

22. The teachers are well prepared for the teaching sessions *

Mark only one oval.

	1	2	3	4	5
	⬭	⬭	⬭	⬭	⬭

23. I feel I am being well prepared for my profession *

Mark only one oval.

	1	2	3	4	5
	⬭	⬭	⬭	⬭	⬭

24. My internet connection can be problematic *

Mark only one oval.

	1	2	3	4	5
	⬭	⬭	⬭	⬭	⬭

25. I had problems with technology to participate efficiently in online education *

Mark only one oval.

1	2	3	4	5
⬭	⬭	⬭	⬭	⬭

26. What aspects of online learning do you enjoy? *

Check all that apply.

- [ ] No travel
- [ ] Ability to ask questions
- [ ] cost savings
- [ ] more comfortable
- [ ] interactive
- [ ] ability to learn at own pace
- [ ] flexible
- [ ] none

27. What do you feel are the barriers to online learning? *

Check all that apply.

- [ ] Internet connection
- [ ] timing of tutorials
- [ ] family or home distractions
- [ ] lack of space
- [ ] lack of devices
- [ ] harder to commit to than face to face learning
- [ ] less communication than face to face learning
- [ ] none

Impact on academic performance and anxiety levels

28. Does studying through online teaching during the covid pandemic affected your academic performance? *

Mark only one oval.

- ( ) Yes, It helped me get better grades than before the pandemic
- ( ) No, It didn't help me get better grades than before the pandemic
- ( ) Neutral. I got the same grades than before the pandemic

29. what is the effect of Online teaching on your anxiety levels on a scale from 1 - 5? (1 = no anxiety, 5 = maximum anxiety) *

Mark only one oval.

1	2	3	4	5
( )	( )	( )	( )	( )

This content is neither created nor endorsed by Google.

Google Forms
